# Test agreement between serum and milk antibody enzyme-linked immunosorbent assay for *Salmonella* Dublin: A cross-sectional diagnostic accuracy study

**DOI:** 10.3168/jdsc.2026-1099

**Published:** 2026-07-27

**Authors:** Christina Lyn, Amy L. Greer, Ángel Abuelo, Chelsea Himsworth, Stephen J. LeBlanc, David L. Renaud

**Affiliations:** 1 Department of Population Medicine, Ontario Veterinary College, University of Guelph, Guelph, ON N1G 2W1, Canada; 2 Department of Biology, Trent University, Peterborough, ON K9L 0G2, Canada; 3 Department of Large Animal Clinical Sciences, Michigan State University, East Lansing, MI 48824; 4 School of Population and Public Health, University of British Columbia, Vancouver, BC V6T 1Z3, Canada

## Abstract

There is increased interest in *Salmonella* Dublin surveillance on dairy farms using either milk or serum samples from individual lactating cows. In practice, sample selection is often based on convenience, and results from milk and serum are frequently used interchangeably. The objective of this cross-sectional diagnostic accuracy study was to evaluate agreement between milk and serum results from individual lactating cows using a commercial antibody ELISA (PrioCHECK S. Dublin Strip Kit; Thermo Fisher Scientific). Milk and serum were collected from 422 lactating cows on 6 dairy farms in British Columbia, Canada. Agreement between milk and serum ELISA percent positivity (PP) was assessed using Lin's concordance correlation coefficient (CCC) and Bland–Altman analysis. Categorical agreement was evaluated using Cohen's kappa, and Youden's index used to identify optimal cut points. There was moderate agreement between milk and serum PP values (CCC = 0.67, 95% CI 0.63–0.71). Using milk at a reference cut point of 35 PP, the optimal serum cut point identified by Youden's index was 1.44 PP, yielding sensitivity of 87.57% and specificity of 89.24%. In contrast, when serum at 35 PP was used as the reference, the optimal cut point for milk was 58.84 PP, producing sensitivity of 95.12% and specificity of 90.29%. Cohen's kappa coefficients indicated fair agreement using a cut point of 35 PP for both milk and serum (κ = 0.36, 95% CI 0.28–0.45), moderate agreement using a milk cut point of 35 PP with serum cut points of 15 PP (κ = 0.57, 95% CI 0.48–0.66) or 1.4 PP (κ = 0.61, 95% CI 0.50–0.65), and substantial agreement using a milk cut point of 58.84 PP and a serum cut point of 35 PP (κ = 0.74, 95% CI 0.67–0.81). A mixed linear regression model was used to investigate the effects of lactation number, DIM, and SCC at sampling on the difference between milk and serum PP, with herd as a random effect. Compared with cows in their first lactation, cows in lactation ≥3 had larger discrepancies between paired PP (β = 6.15; 95% CI 0.65–11.65). Larger differences between milk and serum PP were also associated with SCC between 200,000 and 400,000 cells/mL (β = 11.80, 95% CI 4.92–18.67) and >400,000 cells/mL (β = 8.51, 95% CI 1.82–15.21) compared with SCC ≤100,000 cells/mL. The results suggest that milk and serum ELISA should not be used interchangeably and that meaningful agreement depends on the cut points applied.

## Graphical Abstract

**Figure:**
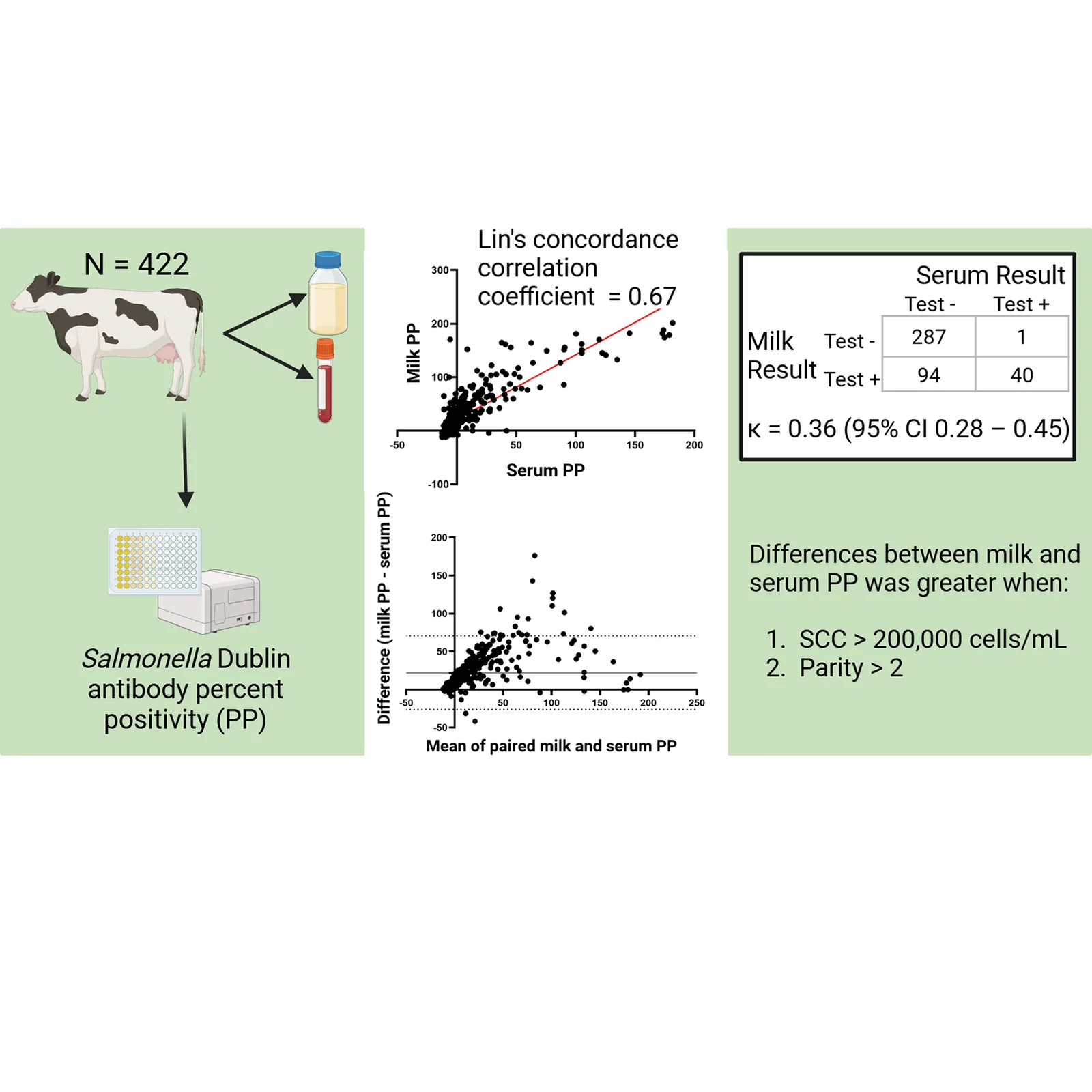

*Salmonella enterica* ssp. *enterica* serovar Dublin (*Salmonella* Dublin) is a gram-negative bacterium that is host-adapted to cattle (Mohler et al., 2009; Holschbach and Peek, 2018; Peek et al., 2018). Young calves are at the greatest risk of clinical disease and often present with septicemia or respiratory disease, or both, refractory to treatment (Mohler et al., 2009; Holschbach and Peek, 2018; Peek et al., 2018). Less commonly, in adult cattle, *Salmonella* Dublin targets the gastrointestinal and reproductive systems, resulting in diarrhea or abortion (Hall and Jones, 1976, 1977; Holschbach and Peek, 2018). Although *Salmonella* Dublin is a host-adapted serovar, it poses a zoonotic threat and is increasingly associated with multidrug resistance, underscoring the importance of surveillance and monitoring programs for both veterinary and public health (Nielsen et al., 2021).

Identification of *Salmonella* Dublin in live animals is primarily based on antibody ELISA testing. The PrioCHECK S. Dublin Ab Strip Kit (Thermo Fisher Scientific) is the main commercially available indirect ELISA used in Canada and the United States. This assay can be performed on serum or milk (individual or bulk tank) and detects antibodies against *Salmonella* Dublin LPS O antigens 1, 9, and 12 (Thermo Fisher Scientific, PrioCHECK S. Dublin Ab Strip Kit unpublished validation report, https://www.thermofisher.com/order/catalog/product/7610640). For individual animal testing, serum ELISA are commonly used, but their performance varies depending on animal age and infection dynamics (Nielsen and Ersbøll, 2004). Using a cut point of 35% positivity (**PP**), Nielsen and Ersbøll (2004) reported sensitivity of 79% (95% CI 62%–91%) and specificity of 98.5% (95% CI 96%–100%) in heifers 100 to 300 d of age. In animals >300 d of age, sensitivity declined to 62% (95% CI 46%–75%) with a specificity of 94% (95% CI 92%–96%) when compared against fecal culture (Nielsen and Ersbøll, 2004).

Individual milk samples have become an increasingly valuable alternative for testing because they allow for noninvasive, repeated sampling and can be easily integrated into routine milk recording systems (Ohlson et al., 2014). When fecal culture was used as the gold standard, individual milk and serum ELISA were reported to have similar sensitivity and specificity (Nielsen, 2003). At a cut point of 25 PP, the sensitivity and specificity were estimated to range from 77% to 78% and from 65% to 86%, respectively (Nielsen, 2003). However, increasing the cut point to 50 PP reduced sensitivity to 42% to 43% while increasing specificity to 81% to 94% (Nielsen, 2003).

Herds infected with *Salmonella* Dublin may benefit from culling of chronically infected animals, typically requiring 3 positive samples over a period of 120 to 240 d to identify carriers (Nielsen et al., 2004; Bergevoet et al., 2009; Henderson and Mason, 2017). Whereas it is commonly assumed that serum and milk ELISA results can be used interchangeably, such that a cow test-positive on serum will also be test-positive on milk, this assumption has not been directly evaluated in lactating cows. Poor agreement between sample types could result in misclassification, with animals either culled despite being uninfected or retained despite being carriers that perpetuate disease transmission. Clarifying the relationship between these 2 sample types will improve diagnostic interpretation and support more accurate surveillance and control for *Salmonella* Dublin. Therefore, the objective of this cross-sectional diagnostic accuracy study was to measure agreement between milk and serum results from individual animals using a commercial antibody ELISA test for *Salmonella* Dublin.

In this prospective study, samples were collected from cows on 6 dairy farms in southern British Columbia, Canada, from December 2023 to March 2024 in accordance with the University of Guelph Animal Care Committee requirements (Animal Use Protocol #5120). Farms were eligible for inclusion if they had 2 consecutive *Salmonella* Dublin positive bulk tank milk samples collected ≥4 mo apart and milked more than 50 cows. All participating farms raised their own replacement heifers but were not closed herds.

A formal sample size calculation was not performed. Instead, the maximum sample size collected per farm was determined based on the available funds for sampling and testing. Approximately 50% of the lactating herd, or up to 75 cows, whichever was smaller, was selected using random sampling stratified by parity. To do this, a list of all lactating animals' identification numbers, parity, and DIM was generated using the farm's record management software. The proportion of the lactating herd in parity 1, 2, and 3 or more was calculated and multiplied by the sample size to determine the number of cows to be sampled in each lactation group. In Microsoft Excel (Microsoft Corporation, Redmond, WA), individual animal identifications were grouped by parity as above and added to a numbered list, which was referenced in Excel's random number generator function to select animals to be sampled. Clinically healthy cows and those with clinical mastitis were included in the study; however, milk from abnormal quarters was excluded from composite samples. Previous *Salmonella* Dublin test status of individual cows was not known.

A composite milk sample and a blood sample were taken <12 h apart in lactating cows. To do this, a 20-gauge, 25-mm Vacutainer needle (BD Vacutainer PrecisionGlide) was used to draw 10 mL of blood from the coccygeal vessels into a silicone-coated tube with no additive (Monoject Blood Collection Tube). Blood was allowed to clot before centrifugation at 1,000 × *g* for 20 min at 15°C. Serum was separated into two 5 mL silicone-coated red top tubes with no additive (Monoject Blood Collection Tube) and frozen at −20°C. The milk sample was taken following stripping and cleaning of the teats with a predip if done in the parlor during milking or stripping and disinfecting with an alcohol swab if samples were taken outside of the parlor. An approximately equal volume of milk from each quarter was added into a 40 mL sterile milk vial. Milk samples were separated into 2 sterile milk vials, frozen, and stored at −20°C.

Paired serum and milk samples were submitted to the Animal Health Center in Abbotsford, British Columbia, and tested for *Salmonella* Dublin antibodies using a commercially available antibody indirect ELISA (PrioCHECK S. Dublin Ab Strip Kit, Thermo Fisher Scientific). Following the manufacturer's recommendations, whole milk samples were added undiluted to the wells, and serum was diluted 20-fold with a buffer solution provided in the test kit (manufacturer's proprietary formulation) before being added to the wells and incubated for 60 min (PrioCHECK S. Dublin Ab Strip Kit, Thermo Fisher Scientific). Plates were then washed before the addition of conjugate and incubated again for 60 min. Last, following another wash, chromogen (3,3′,5,5′-tetramethylbenzidine) substrate was added to the wells and incubated for 15 min before the addition of the stop solution provided in the test kit. The optical density (**OD**) was read at 450 nm with an absorbance spectrophotometer, and results were reported as PP relative to a positive control calculated according to the following formula:

$$\text{\textbackslash{}[}PP=\frac{\left(OD_{sample}-mean\;OD_{negative\;control}\right)}{\left(OD_{positive\;control}-mean\;OD_{negative\;control}\right)}\times 100\%-10.\text{\textbackslash{}]}$$

For the test to be valid, the mean OD of the negative controls must have been <0.4, the positive control >1 and the validation control PP ≥30. Technicians were not blinded with respect to serum and milk sample ELISA results.

The second milk sample was sent to the Pacific Milk Analysis Laboratory in Chilliwack, British Columbia, for SCC analysis. The SCC was determined via flow cytometry using a Fossomatic 7 (Foss Analytical A/S), with results reported as cells per milliliter. Freezing milk samples before analysis has been shown to have minimal impact on SCC when measured using fluoro-opto-electronic cell counters (Barkema et al., 1997).

Data were imported from Excel (Microsoft Corporation, Redmond, WA) into STATA 19.5 (StataCorp LP) for statistical analysis. The results were initially investigated using McNemar and Pearson chi-squared tests at a 35 PP cut point. The McNemar test was used to compare paired serum and milk ELISA results and assess whether serum and milk results differed by evaluating discordant pairs (i.e., animals positive on one test but negative on the other). A Pearson chi-squared test was then used to evaluate whether the distribution of test results differed among lactation groups for both serum and milk.

A Bland–Altman plot was used to assess agreement between serum and milk PP by plotting the difference between paired measurements against their mean. The plot visualizes bias and limits of agreement, evaluating whether the 2 methods can be used interchangeably. Agreement between PP was also evaluated using Lin's concordance correlation coefficient (**CCC**). A value of −1 represents perfect disagreement, and a value of 1 represents perfect agreement. Lin's CCC was interpreted as follows: 0.00 to 0.30 = negligible, 0.30 to 0.50 = low, 0.50 to 0.70 = moderate, 0.70 to 0.90 = high, and 0.90 to 1.00 = very high (Hinkle, 1988). Cohen's kappa was calculated to assess the categorical agreement between milk and serum results at the manufacturer's cut point of 35 PP and at 15 PP, which is used in some laboratories (Um et al., 2022; Nobrega et al., 2024). Interpretation of Cohen's kappa was based on established thresholds, where 0 indicates no agreement, 0.01 to 0.20 indicates none to slight, 0.21 to 0.40 fair, 0.41 to 0.60 moderate, 0.61 to 0.80 substantial, and 0.81 to 1.00 almost perfect agreement (McHugh, 2012).

To identify cut points that maximize agreement between samples, 2 receiver operating characteristic (**ROC**) curve analyses were conducted. As neither sample (milk nor serum) is a gold standard reference test, improved diagnostic accuracy cannot be inferred. One ROC curve compared milk classification at 35 PP to serum PP, and the other compared serum classification of *Salmonella* Dublin status using 35 PP to milk PP to identify cut points that maximized sensitivity and specificity based on the Youden index. The area under the curve (**AUC**) was used to quantify overall diagnostic accuracy, with higher values indicating better discrimination between positive and negative cases. The AUC values were interpreted as follows: 0.5 to 0.6 as fail, 0.6 to 0.7 as poor, 0.7 to 0.8 as fair, 0.8 to 0.9 as considerable, and ≥0.9 as excellent diagnostic accuracy (Çorbacıoğlu and Aksel, 2023). Cohen's kappa was also calculated based on the cut points calculated using Youden's index.

A mixed linear regression model, with farm as a random effect, was used to investigate factors associated with the difference between milk and serum PP. Fixed effects considered in the model included lactation number (1, 2, or ≥3), DIM, and SCC. Somatic cell count did not meet the linearity assumption and was categorized into 4 groups: ≤100,000 cells/mL, 100,000 to 200,000 cells/mL, 200,000 to 400,000 cells/mL, and >400,000 cells/mL. Each variable was screened separately, and those with a *P*-value ≤0.20 were included in the multivariable model. Using backward selection, variables with the largest *P*-value were removed sequentially. A variable was deemed a confounder if removal resulted in a 20% change in a remaining coefficient. Because only 3 variables were included in the model, all possible 2-way interaction terms were examined. Variables remained in the model if their *P* ≤ 0.05. Homoscedasticity and normality of the residuals and BLUP were visually assessed to ensure model fit.

A total of 422 lactating cows were sampled, with 75 cows sampled on farms 1, 2, 4, and 5, 49 on farm 3, and 73 cows on farm 6. Cows were sampled in lactations one through 8, including 148 in first lactation, 124 in second, and 150 in lactation ≥3. DIM at sampling ranged from 1 to 628, with a median of 157 d. A total of 415 samples were analyzed for SCC, as 7 samples did not have a large enough volume of milk to be tested. The SCC ranged from 11,000 to 14,562,000 cells/mL, with a median SCC value of 91,000 cells/mL.

At the manufacturer-recommended cut point of 35 PP, milk and serum ELISA classifications differed (*P* < 0.001). Based on milk ELISA results, 32% of cows were classified as positive compared with 10% classified as positive using serum ELISA. For farms 1 through 6, the proportion of cows positive on milk ELISA was 31%, 48%, 12%, 37%, 23%, and 33%, respectively. In contrast, the proportion of cows positive on serum ELISA for farms 1 through 6 was 7%, 15%, 2%, 12%, 12%, and 8%, respectively. The proportion of cows classified as positive did not differ by lactation group for milk (*P* = 0.83) or serum (*P* = 0.26).

Among the 422 paired samples, 327 were concordant and 95 were discordant. All but one discordant classification occurred when samples were categorized as positive on milk and negative on serum. Bland–Altman analysis (Figure 1) demonstrated a mean difference of 22 PP units, with higher values observed in milk than serum, indicating systematically greater values in milk. Paired milk and serum PP values showed a positive linear association (Figure 2), with Lin's CCC of 0.67 (95% CI 0.63–0.71), indicating moderate agreement.

**Figure 1:**
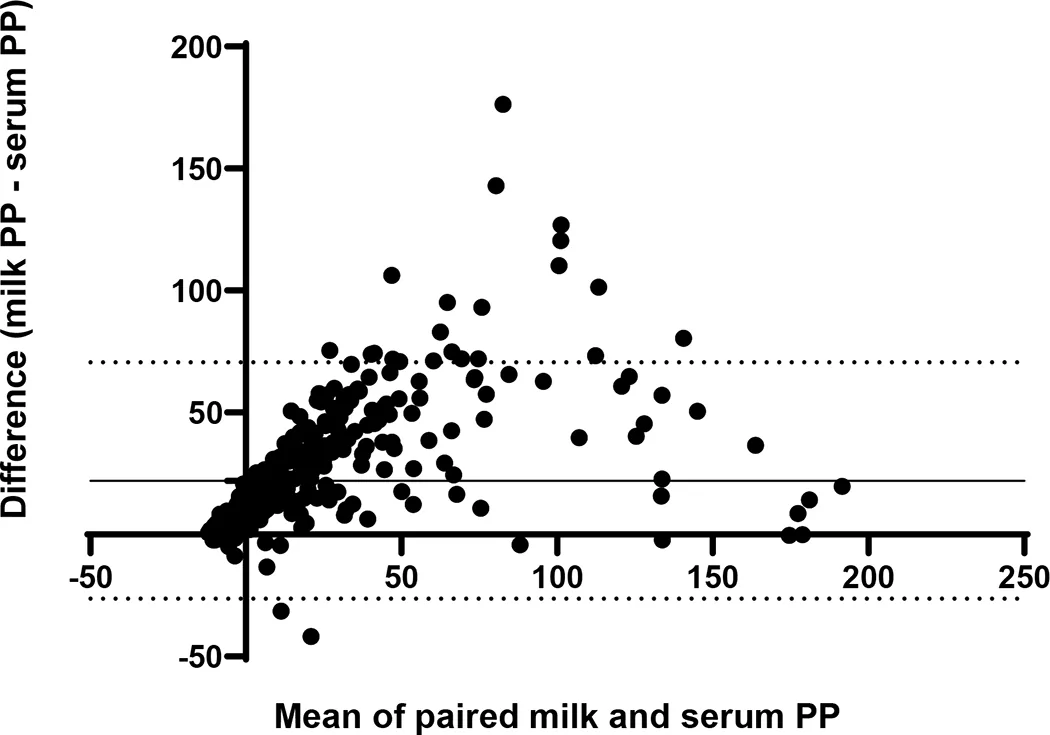
Bland–Altman plot illustrating agreement between 422 paired milk and serum ELISA (PrioCHECK S. Dublin Strip Kit; Thermo Fisher Scientific) results (expressed as percent positivity [PP]), with differences calculated as milk PP minus serum PP. The solid line indicates the mean difference (mean = 22), and dashed lines represent the 95% limits of agreement at −22 and 71.

**Figure 2:**
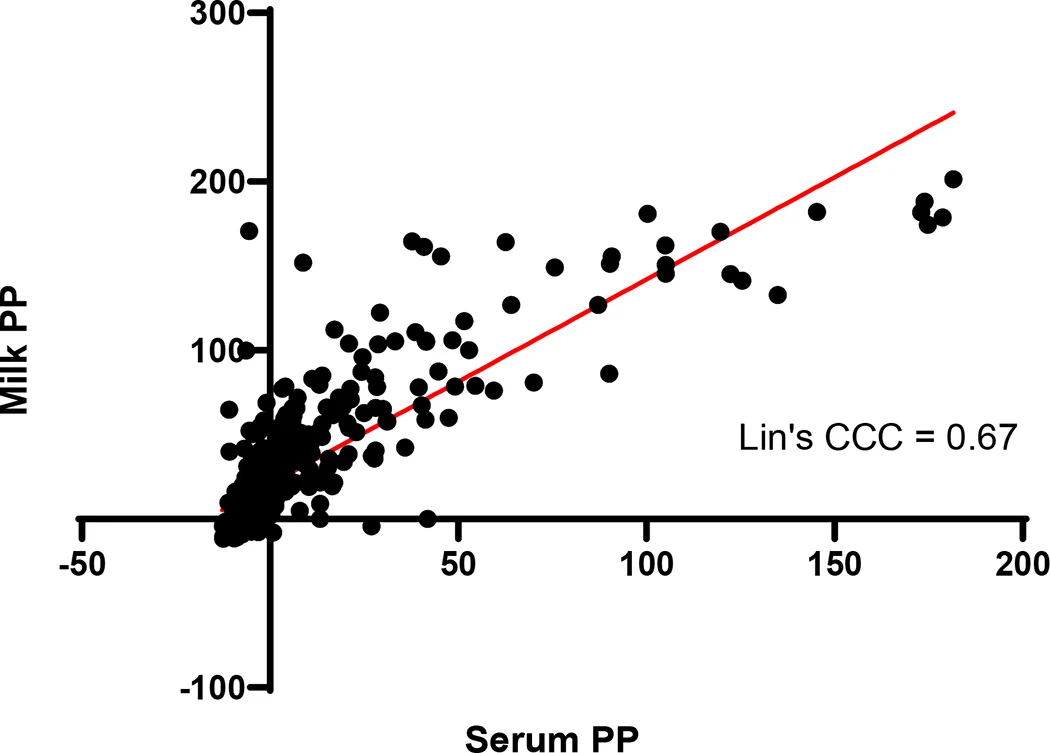
Scatterplot comparing percent positivity (PP) between 422 paired serum and milk *Salmonella* Dublin antibody ELISA (PrioCHECK S. Dublin Strip Kit; Thermo Fisher Scientific) results; the relationship was quantified using Lin's concordance correlation coefficient = 0.67 (95% CI 0.63–0.71).

Using milk results at a cut point of 35 PP as the reference, a ROC analysis was conducted to identify the ideal cut point of serum PP, which would result in the greatest agreement. The area under the curve was 0.93 (95% CI 0.90–0.96), indicating excellent discriminatory ability. The optimal serum cut point identified using the Youden index was 1.44 PP, yielding sensitivity of 86.57%, specificity of 89.24%, positive predictive value of 78.80%, and negative predictive value of 93.10%. A second ROC analysis, using serum results at a cut point of 35 PP as the reference, also demonstrated excellent discrimination, with an area under the curve of 0.96 (95% CI 0.93–1.00). The optimal milk cut point determined by the Youden index was 58.84 PP, yielding sensitivity of 95.12%, specificity of 90.29%, positive predictive value of 51.30%, and negative predictive value of 99.40%.

Categorical agreement between serum and milk at the manufacturer-recommended cut point of 35 PP cut point was fair, with a Cohen's kappa value of 0.36 (95% CI 0.28–0.45). Reducing the serum cut point to 15 PP, while maintaining the milk cut point at 35 PP, improved agreement to moderate, with a Cohen's kappa value of 0.57 (95% CI 0.48–0.66). Reducing the serum cut point to 1.44 PP, as identified by ROC analysis, while maintaining the milk cut point at 35 PP resulted in substantial agreement, with a Cohen's kappa value of 0.74 (95% CI 0.67–0.81). Conversely, increasing the milk cut point to 58.84 PP, as identified by ROC analysis, while maintaining the serum cut point at 35 PP resulted in substantial agreement, with a Cohen's kappa value of 0.62 (95% CI 0.51–0.73).

The final mixed linear regression model evaluating differences in PP between milk and serum identified associations with lactation group and SCC. The difference between paired milk and serum PP was greatest in cows in lactation ≥3 (β = 6.15; 95% CI 0.65–11.65; *P* = 0.029) relative to those in their first lactation. For SCC, values of 200,000 to 400,000 cells/mL (β = 11.80, 95% CI 4.92–18.67, *P* = 0.001) or >400,000 cells/mL (β = 8.51, 95% CI 1.82–15.21, *P* = 0.01) were also associated with an increased PP difference between paired milk and serum samples, compared with cows with SCC ≤100,000 cells/mL.

Our results demonstrate that agreement between paired milk and serum *Salmonella* Dublin antibody ELISA results in lactating cows is limited when both sample types are interpreted using the manufacturer's recommended cut point of 35 PP. This indicates that milk and serum results should not be assumed to be directly interchangeable. Importantly, a positive linear relationship between milk and serum PP values was observed, highlighting that agreement can be improved through adjustments of cut points for either milk or serum, depending on the intended application.

A recent study investigating agreement between milk and serum using 2 *Mycoplasma avium* subspecies *paratuberculosis* (**MAP**) ELISA tests reported the within-test agreement of serum and milk to be moderate or substantial (κ = 0.55, 95% CI: 0.46–0.65 and κ = 0.79, 95% CI: 0.71–0.88; Venkatesh et al., 2025). Both MAP ELISA tests used cut points specific to the sample tested (i.e., milk or serum; Venkatesh et al., 2025). The lower agreement observed in the present study supports the idea that the cut points could be adjusted to achieve better agreement. Differences in optimal cut points between milk and serum may reflect variation in infection dynamics, immune responses, or *Salmonella* Dublin antibody partitioning into milk.

Methodological factors such as sample processing should also be considered. In general, Ig concentrations differ between serum and milk, with higher concentrations in serum (Butler et al., 2015). This physiological difference between serum and milk provides a rationale for diluting serum by a factor of 20 and testing milk undiluted, as done here; however, the validation report provided by the manufacturer of the PrioCHECK S. Dublin Ab Strip Kit does not explain how the dilution factor for serum was determined (Thermo Fisher Scientific, PrioCHECK S. Dublin Ab Strip Kit unpublished validation report, https://www.thermofisher.com/order/catalog/product/7610640). The optimal serum dilution should be further investigated with respect to its influence on test agreement.

Nielsen and Toft (2012) recommend excluding cows 1 to 5 DIM to avoid the effects of colostrum and transition milk. In the present study, only 9 cows were <6 DIM, and their exclusion had minimal impact on agreement. However, discrepancies were noted between milk and serum results in cows in their third or greater lactation. A dilution effect, whereby high milk yield reduces antibody concentration despite relatively constant IgG secretion, has been reported to result in false-negative MAP milk ELISA tests (Nielsen and Toft, 2012; Eisenberg et al., 2015). Although milk yield was not measured in the present study, dilution of antibodies might explain the lesser agreement between milk and serum ELISA results in higher-parity cows.

Milk quality has also been implicated in false-positive milk ELISA results. (Klintevall et al., 1991) reported that 4 of 11 cows with clinical mastitis produced false-positive BLV ELISA results in milk from the affected quarter, whereas paired serum samples and milk from unaffected quarters tested negative. In the current study, quarters with clinical mastitis were excluded; however, greater SCC categories were associated with an increased discrepancy between milk and serum PP. Further research is warranted to determine whether SCC-based correction factors could improve agreement between milk and serum results.

There are a few limitations to consider when interpreting the results of this study. Herds were purposefully selected based on prior evidence of *Salmonella* Dublin infection to ensure sufficient seropositive animals for comparison of paired milk and serum ELISA results. As a result, these herds may not be representative of the broader dairy population. In addition, confirmatory diagnostic testing, such as fecal culture or PCR, was not performed. Because no independent gold standard is available for comparison, diagnostic accuracy could not be directly assessed. Consequently, the cut points identified in this study should be interpreted as relative thresholds that improve agreement between milk and serum results rather than as optimized diagnostic cut points for infection status. Future research should aim toward establishing standardized cut points to improve *Salmonella* Dublin diagnosis.

This study evaluated agreement between paired milk and serum samples from lactating cows using a commercial antibody ELISA, finding moderate agreement between serum and milk PP and fair categorical agreement of positive and negative animals. The categorical agreement could be improved by adjusting the cut point by either increasing the milk cut point or reducing the serum cut point. Higher SCC and parity were associated with increased differences in paired milk and serum PP, therefore affecting agreement. Our results suggest that serum and milk ELISA should not be used interchangeably and that interpretation of test results or selection of sample type should be guided by the specific surveillance objective.
